# Latency and rate coding in a spiking model of retina

**DOI:** 10.1186/1471-2202-14-S1-P186

**Published:** 2013-07-08

**Authors:** D Fisher, C Petre, B Szatmáry, M Buibas, E Izhikevich

**Affiliations:** 1 Brain Corporation, San Diego, CA, USA

## 

We describe a family of spiking retina computational models for large-scale simulations of the visual pathways. The models reproduce the overall spatial, temporal, and chromatic structure of the receptive fields of midget and parasol retinal ganglion cells (RGCs) of the primate retina, as well as their contrast response, while using fairly simple models of bipolar, horizontal, amacrine, and RGCs. These retina models provide input to a realistic model of visual cortex, presented in a separate submission.

**Figure 1:**
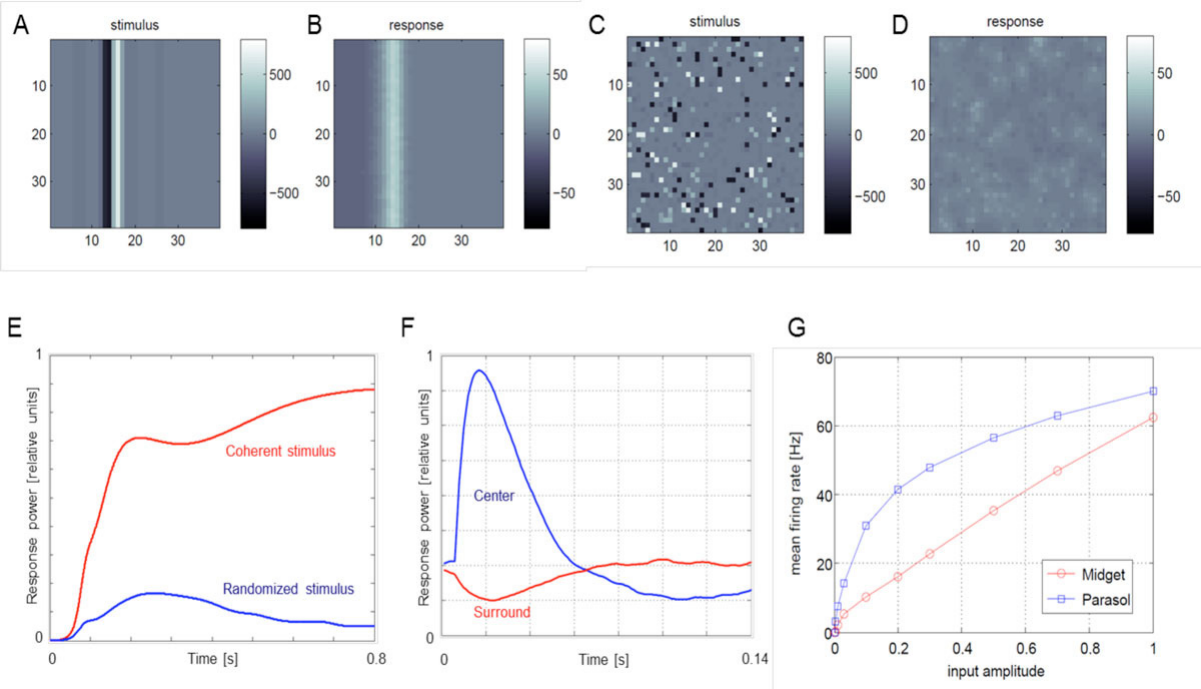
**A-E: model response to coherent vs. randomized stimulus**. Moving edge stimulus is presented (A) and elicits strong response (B). Same stimulus with fixed random permutation of pixels (C) elicits much weaker response (D). (E) compares the total responses in B (red) and D (blue). (F) shows reverse-correlated time courses of the center (blue) and surround (red) inputs to a parasol cell. (G) shows spiking responses of parasol (saturating, blue) and midget cells (linear, red) to input strength.

The retina model simulates cone and bipolar cell dynamics using a damped wave equation. Parameters are so chosen that an impulse retinal input gives rise to a damped biphasic output current, and that a coherent stimulus elicits a substantially stronger response than an incoherent stimulus of the same power (Figure 1). Static or low-frequency visual inputs are smoothly rejected. In contrast to biology, this stage of the model is linear; rectification and adaptation mechanisms are all realized in the spiking ganglion cell layer. In the ganglion cell layer parasol, midget, and SBC cells are simulated. The spatio-temporal receptive fields of different RGCs are not prewired; they emerge via the lateral interactions between the horizontal, bipolar, and amacrine cells. Spike generation in the RGCs is modeled using spiking neurons with two dynamic variables and an intrinsic adaptation mechanism. Midget RGCs have an additional leaky temporal integration of the input current, to account for the relatively tonic character of their responses. Parasol RGCs have an additional partial divisive normalization of the input current, to reproduce the saturating parasol response to relative contrast of the stimulus. Simplified amacrine-cell dynamics is introduced to provide for the spatiotemporal structure of the parasol receptive field surrounds.

Desired latency encoding of input features is achieved by calibrating the parameters of the spiking RGC conductance dynamics. A near logarithmic latency to the first spike, for a step up in the input signal, provides for a good contrast invariance of the relative spike timing in the response. A combination of spike-latency and spike-count encoding of the input stimulus, together with the enhanced response of the simulated retina to coherent stimuli, results in an informative yet not too noisy spiking input to the LGN and V1; the information content analysis of the RGC spike-train vocabulary will be presented. This retina model was used to produce a fully-emergent orientation tuning in a spiking model [1] of V1 cortex.
